# Replication of Genome Wide Association Studies of Alcohol Dependence: Support for Association with Variation in *ADH1C*

**DOI:** 10.1371/journal.pone.0058798

**Published:** 2013-03-13

**Authors:** Joanna M. Biernacka, Jennifer R. Geske, Terry D. Schneekloth, Mark A. Frye, Julie M. Cunningham, Doo-Sup Choi, Courtney L. Tapp, Bradley R. Lewis, Maureen S. Drews, Tracy L.Pietrzak, Colin L. Colby, Daniel K. Hall-Flavin, Larissa L. Loukianova, John A. Heit, David A. Mrazek, Victor M. Karpyak

**Affiliations:** 1 Department of Psychiatry & Psychology, Mayo Clinic, Rochester, Minnesota, United States of America; 2 Division of Biomedical Statistics & Informatics, Department of Health Sciences Research, Mayo Clinic, Rochester, Minnesota, United States of America; 3 Department of Laboratory Medicine & Pathology, Mayo Clinic, Rochester, Minnesota, United States of America; 4 Department of Molecular Pharmacology & Experimental Therapeutics, Mayo Clinic, Rochester, Minnesota, United States of America; 5 Department of Information Technology, Mayo Clinic, Rochester, Minnesota, United States of America; 6 Department of Internal Medicine, Mayo Clinic, Rochester, Minnesota, United States of America; Sanjay Gandhi Medical Institute, India

## Abstract

Genome-wide association studies (GWAS) have revealed many single nucleotide polymorphisms (SNPs) associated with complex traits. Although these studies frequently fail to identify statistically significant associations, the top association signals from GWAS may be enriched for true associations. We therefore investigated the association of alcohol dependence with 43 SNPs selected from association signals in the first two published GWAS of alcoholism. Our analysis of 808 alcohol-dependent cases and 1,248 controls provided evidence of association of alcohol dependence with SNP rs1614972 in the *ADH1C* gene (unadjusted p = 0.0017). Because the GWAS study that originally reported association of alcohol dependence with this SNP [1] included only men, we also performed analyses in sex-specific strata. The results suggest that this SNP has a similar effect in both sexes (men: OR (95%CI) = 0.80 (0.66, 0.95); women: OR (95%CI) = 0.83 (0.66, 1.03)). We also observed marginal evidence of association of the rs1614972 minor allele with lower alcohol consumption in the non-alcoholic controls (p = 0.081), and independently in the alcohol-dependent cases (p = 0.046). Despite a number of potential differences between the samples investigated by the prior GWAS and the current study, data presented here provide additional support for the association of SNP rs1614972 in *ADH1C* with alcohol dependence and extend this finding by demonstrating association with consumption levels in both non-alcoholic and alcohol-dependent populations. Further studies should investigate the association of other polymorphisms in this gene with alcohol dependence and related alcohol-use phenotypes.

## Introduction

Alcohol dependence is known to be under considerable genetic influence [2], [3], yet few genetic risk factors have been discovered and confirmed. Genome-wide association studies (GWAS) have successfully identified many single nucleotide polymorphisms (SNPs) associated with complex traits. However, GWAS generally have low power to detect individual SNP effects. Thus, many of these studies fail to identify associations that are statistically significant at the genome-wide level. Nevertheless, the top association signals from GWAS are expected to be enriched for true associations and provide good candidates for further follow-up. Replication of the top GWAS signals in independent data sets is therefore an important strategy for identifying genetic factors contributing to complex traits.

The first GWAS of alcohol dependence was published by Treutlein and colleagues [1]. Stage 1 of this study consisted of a GWAS based on 487 early age-of-onset alcohol-dependent male cases and 1,358 controls. Results of this analysis were integrated with findings from animal model studies of candidate genes to select SNPs for further follow-up. The follow-up study included 121 SNPs that produced p-values <10^−4^ in the GWAS, as well as 19 nominally significant SNPs from genes of which homologs had shown expression changes in rat brains in response to alcohol consumption. The SNPs selected for follow-up were genotyped in an additional 1,024 cases and 996 controls. The follow-up study identified 15 SNPs that showed nominally significant association with the same allele as in the GWAS, including two intergenic SNPs with genome-wide significance. The 15 SNPs included SNPs in the Cadherin 13 (*CDH13)* and the alcohol dehydrogenase 1C (*ADH1C*) genes, which have been previously reported to be associated with alcohol dependence. In particular, variation in the alcohol dehydrogenase gene cluster is a consistently replicated contributor to alcohol-related phenotypes [2], [4].

Subsequently, Bierut et al. [5] published a GWAS of alcohol dependence based on the Study of Addiction, Genetics and Environment (SAGE). This study included 1,897 European-American and African-American subjects with alcohol dependence and 1,932 independent controls. Fifteen SNPs with p<10^−5^ were identified in the GWAS; however, in two independent replication samples none of the SNPs passed the statistical significance threshold of p<0.05.

Here we report the results of a replication study of association of alcohol dependence focused on 43 SNPs selected on the basis of association signals in the first two published GWAS of alcohol dependence [1], [5]. For SNPs for which we obtained replicated evidence of association with alcohol dependence, we also investigated association with alcohol consumption level.

## Methods

### Ethics Statement

This study was approved by the Mayo Clinic Institutional Review Board. All subjects provided written informed consent for use of their DNA in research.

### Subjects

Our analysis included 808 alcohol-dependent cases and 1,248 non-alcohol-dependent controls. The cases included subjects from Mayo Clinic’s DNA Repository for Genomic Studies of Addiction (272 from the residential Intensive Addiction Program and 137 from the Outpatient Addiction Program), 97 subjects from a liver transplant study, and 343 subjects from ongoing studies of genetic predictors of severe alcohol withdrawal [6], [7]. All cases were evaluated by a board certified psychiatrist, and only subjects meeting DSM-IV diagnostic criteria for alcohol dependence were included in the study. For the alcohol withdrawal studies, in addition to establishing a diagnosis of alcohol dependence, a standardized questionnaire (a modified version of Lifetime Drinking History) was used to collect alcohol consumption data, including average number of drinks per drinking day measured in units of standard drinks, with standard drink defined according to the National Institute on Alcohol Abuse and Alcoholism (NIAAA) as 14 g of alcohol. Alcohol consumption measures for the inpatient and outpatient addiction programs and the transplant study were not uniformly collected using standardized measures.

For cost efficiency, previously genotyped controls were used for this study. Controls were selected from a group of controls included in a GWAS of venous thrombosis carried out at Mayo Clinic [8], [9]. Thus the controls had been previously selected to have no history of venous thrombosis, but were otherwise representative of the general population. From the group of 1,302 genotyped controls, 15 subjects with a documented history of alcohol dependence were excluded.

### SNP Selection

Candidate SNPs were selected based on published results of the German GWAS reported by Treutlein et al. [1], as well as our analysis of data from the Study of Addiction: Genetics and Environment (SAGE) [5]. Because these studies did not detect SNP associations that were significant at the genome-wide level (p<5*10^−8^), we used less stringent criteria for selection of SNPs for our study, recognizing that the top results in a GWAS are likely to harbor some true associations that did not quite reach the stringent criteria for genome-wide significance. For the study of Treutlein et al., we selected SNPs that were replicated within the study (p<0.05 in the replication stage) and SNPs with p<10^−6^ in the GWAS (discovery) stage. We also selected SNPs with p<10^−4^, in genes where two or more SNPs in low to moderate linkage disequilibrium (LD) (r^2^<0.7), had p<10^−4^ in the GWAS stage of the study. Finally, for SNPs that were replicated, we also included any additional SNPs in the same gene with p<10^−4^ in this study.

As the SAGE results were not published before we initiated this study, we selected candidate SNPs based on our own analyses of data obtained through the Database for Genotypes and Phenotypes (dbGaP; study accession phs000092.v1.p1) rather than the top associated SNPs reported by Bierut et al. [5]. After excluding non-European-American subjects, we applied the quality control filters for the European-American subject set distributed with the data by the SAGE investigators. Genotypes in specific regions for several individuals were set to missing. These regions were identified as anomalous genotype intensity patterns that may indicate aneuploidy or problems with genotyping. Heterozygous haploid genotypes were also set to missing. The quality control filters also excluded samples with missing call rate ≥2%, and SNPs with missing call rate ≥2%, minor allele frequency (MAF) <1%, or Hardy-Weinberg Equilibrium P-value <10^−4^. SNPs were also removed if there were Mendelian errors in more than one family, or more than one duplicate subject with discordant alleles was observed, as well as if a sex difference in allele frequency ≥0.2 was observed.

After applying these quality control filters established by the SAGE investigators, a further 209 SNPs with MAF <0.01 in the final subject subset were removed, and 1,257 SNPs were removed because of evidence of departure from Hardy Weinberg Equilibrium at the p<0.001 level. Our final data set consisted of 839,409 SNPs for 2,544 white subjects, including 1,165 alcohol-dependent cases and 1,379 controls. Prior to testing for association with alcohol dependence, we analyzed the data using EIGENSTRAT [10] to determine principal components that capture any remaining population stratification among these subjects. We then tested the association of each SNP with alcohol dependence using PLINK [11] with a logistic regression model adjusting for five principal components calculated with EIGENTSTRAT. Based on the results of our genome-wide association analysis of the SAGE data, we selected SNPs with p<10^−5^ for our replication study. We also included SNPs with p<10^−4^ if at least two SNPs in the same gene, with LD r^2^<0.7, had p<10^−4^.

We emphasize that candidate SNPs selected for our study were based on published results of the GWAS performed by Treutlein et al. [1], and on our analysis of the SAGE data, noting that our targets based on the SAGE data are not the top signals reported by Bierut et al. [5]. This is due to differences between the analyses performed by the SAGE study investigators and our group, such as differences in covariate adjustment and methods used to control for population stratification. Small differences in analytical procedures can lead to slight differences in p-values, changing the ranking of the top findings. However, we note that many of the SNPs that we investigated were in genes or regions subsequently found to be associated with alcohol dependence by Zuo et al. [12] in a GWAS using combined data from SAGE and COGA (the Collaborative Study on the Genetics of Alcoholism). The SNPs from our genotyped list that had been detected by the analysis of Bierut et al. [5] or Zuo et al. [12] are indicated in the Table S1 in File S1.

### Genotyping and Quality Control

Controls were previously genotyped at the Center for Inherited Disease Research using the Illumina (San Diego, CA) 660 genome-wide SNP array. From the list of candidate SNPs obtained as described above, 46 SNPs are on the Illumina 660 platform, and had therefore been genotyped in the available controls. These 46 SNPs were therefore selected for genotyping in the cases. In addition to the candidate SNPs, 30 ancestry informative SNPs were genotyped [13]. The genotyped ancestry informative SNPs are listed in Table S2 in File S1 while the replication candidate SNPs are listed in Table S1 in File S1. Cases were genotyped at the Mayo Clinic on the Illumina BeadXpress platform using a VeraCode SNP panel following the manufacturer’s protocol. For quality control, a CEPH family trio (Coriell Institute) was genotyped six times, and DNA from four cases was included in duplicate. Concordance between replicates was 100% and there were no Mendelian inheritance errors. In addition, one of the CEPH controls genotyped with the cases had also been genotyped on the Illumina 660 platform with the control subjects, allowing for a genotype concordance check across genotyping platforms. The genotypes for this subject were 100% concordant across platforms.

Three of the 46 candidate SNPs and three ancestry informative markers failed genotyping in the cases; thus 43 candidate SNPs and 27 ancestry informative markers were successfully genotyped. All remaining markers had call rates >97%, with a mean call rate of 99.6%. One ancestry informative marker (rs7657799) demonstrated strong departures from Hardy Weinberg Equilibrium in the cases (p<0.001).

To avoid false positive findings due to population stratification, analyses were limited to subjects of European ancestry. Thirty alcohol-dependent cases failed genotyping, while 903 were successfully genotyped as part of this study, including 809 that were self-reported Caucasian. After exclusion of control subjects with self-reported race other than Caucasian, 1,249 control subjects remained.

### Statistical Analysis

Subjects with self-reported race other than “Caucasian” were excluded from analyses. The ancestry informative markers were then analyzed using STRUCTURE [14] to verify self-reported race of the 809 cases and 1,249 controls that were self-reported to be Caucasian. To help check the genetic ancestry of our subjects, we included 209 HapMap samples (60 CEU, 60 YRI, and 89 CHB/JPT) in the STRUCTURE analysis, and found that the genotyped ancestry informative SNPs performed well for identifying African (YRI) ancestry, but not as well at distinguishing European (CEU) from Asian (CHB/JPT) ancestry. Probabilities of membership in each of the three known race groups of the HapMap samples were calculated for each of our study subjects. This analysis identified one self-reported Caucasian case and one self-reported Caucasian control that had >30% African (YRI) ancestry (Figure S1 in File S1). These subjects were excluded from association analyses, leading to a total 808 cases and 1,248 controls.

Following the subject and SNP selections described above, the genetic association analyses included data for 808 cases (546 men, 262 women) and 1,248 controls (603 men, 645 women) of European descent, and tested for association of alcohol dependence with 43 candidate SNPs. Likelihood ratio tests from logistic regression models were used to evaluate the association of each SNP with alcohol dependence. Genotypes were coded as 0, 1, 2 representing the minor allele frequency, which fits a model with log-additive allele effects. The analyses were repeated with inclusion of covariates representing the ancestry probabilities determined by the STRUCTURE [14] analysis to adjust for potential effects of population stratification. A Bonferroni correction for multiple testing was applied to the p-values. With this multiple testing correction (i.e., assuming a significance threshold of 0.0012), the sample of 808 cases and 1,248 controls provided 80% power to detect odds ratios of 1.31 for common SNPs with minor allele frequencies of 0.4, or odds ratios of 1.51 for SNPs with minor allele frequency of 0.10. Power calculations were performed using the software QUANTO developed by Gauderman and Morrison (http://hydra.usc.edu/gxe).

For one SNP for which we replicated a finding of association with alcohol dependence, we performed a fixed-effects meta-analysis combining the results of the studies of Treutlein et al. [1], Bierut et al. [5], as well as our study results.

For the one SNP for which we replicated the finding of association with alcohol dependence, we also investigated the association of the SNP with a measure of alcohol consumption. Genotype at the rs1614972 SNP was again coded as 0, 1, 2 representing the number of minor alleles carried by a subject. As the controls were recruited for a study unrelated to alcohol dependence, the data collection protocols were different for the cases and controls, and thus different measures of consumption were available for cases and controls. Therefore association of genotypes with alcohol consumption was assessed separately from the cases, using different statistical models.

For the controls, past and current alcohol consumption was reported in categories of: 2–4 per day, 1 per day, 5–6 per week, 2–4 per week, 1 per week, 1–3 per month, and less than 1 per month. For our analysis of alcohol consumption, we excluded subjects less than 21 years of age and those who reported no consumption in the past or current consumption of less than 1 per month. We then grouped the consumption categories to define an ordinal variable with three levels, representing “rare,” “intermediate,” and “frequent” drinking. One drink per week and 1–3 per month were classified as rare drinking; 2–4 per week was considered intermediate; while 5–6 per week, 1 per day, and 2–4 per day were classified as frequent drinking. Based on this classification, 298 control subjects were rare drinkers, 157 were intermediate drinkers, and 140 were frequent drinkers. Association between the genotype and the ordinal consumption measure was then analyzed using a Spearman partial correlation [15], accounting for age and gender effects.

Because collection of alcohol consumption data differed between the studies that contributed alcohol-dependent cases (see Methods section for details), analyses of consumption data in cases were limited to subjects from the alcohol withdrawal studies, as more reliable consumption data were available for these subjects. In particular, in this subgroup of cases, data had been collected using two quantitative consumption measures: average drinks per drinking day, and maximum drinks consumed in a 24-hour period. The analyses of these quantitative traits were performed using linear regression with log-transformed average drinks per drinking day or lifetime maximum drinks consumed in a 24-hour period as the outcome, taking age and gender effects into account by including these covariates in the regression models. Statistical analyses were conducted primarily using SAS version 9.2 (SAS Institute. Cary. NC); the meta-analysis was performed in R (http://www.R-project.org) using the rmeta package.

## Results

Genetic association analyses for alcohol dependence used data from 808 cases and 1,248 controls. Alcohol-dependent cases had an average age of 49.0±12.1 years and 546 (68%) were male. Controls had an average age of 57.2±15.9 years and 603 (48%) were male.

Table 1 shows the top results from our replication study (SNPs with p<0.10). The strongest evidence of association with alcohol dependence was obtained for SNP rs1614972 in the *ADH1C* gene (OR = 0.80, 95% CI = (0.70, 0.92), p = 0.0017). This association remains marginally significant after Bonferroni correction for multiple testing (Bonferroni-corrected p = 0.074). The result is similar when STRUCTURE [14] ancestry probabilities are included as covariates, to adjust for potential effects of population stratification (p = 0.0015). The results also remain similar, although less significant, when the analysis is adjusted for age and gender (p = 0.019). Our findings are highly consistent with the results of Treutlein et al. [1] as demonstrated by similar odds ratios for the effect of the minor allele (Table 2). In fact, a one-sided test of association evaluating the evidence for an effect in our sample with the same direction as that observed by Treutlein et al. (i.e., test of the alternative hypothesis OR <1) provides evidence of association in our sample that remains significant after Bonferroni correction (p = 0.00084, Bonferroni-corrected p = 0.036).

**Table 1:** SNPs with P<0.1 in our Replication Study.

SNP	Gene	Chr	Reason for Inclusionin Study	Minor allele	MAF cases	MAF controls	OR	P-value	P_adjusted_ 1
rs1614972	ADH1C	4	Replicated in German GWAS	C	0.265	0.311	0.80	0.0017	0.0015
rs708006	MTCH1/PI16	6	German GWAS: p<10^−6^ in discovery stage	C	0.142	0.173	0.79	0.0074	0.0072
rs728115	KCND2	7	SAGE GWAS: 2 SNPs in same gene with p<10^−4^	G	0.108	0.091	1.21	0.073	0.081
rs17142876	KCND2	7	SAGE GWAS: 2 SNPs in same gene with p<10^−4^	A	0.103	0.086	1.21	0.075	0.084

1 P_adjusted_ = P-value from a logistic regression model adjusted for ancestry probabilities determined by STRUCTURE analysis.

**Table 2:** Association of alcohol dependence with *ADH1C* SNP rs1614972 in the study of Treutlein et al. and our replication study, including sex-specific association.

Study	N cases	N controls	MAF cases	MAF controls	OR (95% CI)	P-value
German GWAS (Treutlein et al. [1])						
Stage 1 (GWAS)	476	1358	0.25	0.31	0.73 (0.62, 0.86)	0.00028
Replication	984	974	0.28	0.31	0.86 (0.75, 0.99)	0.036
Combined	1460	2332	0.27	0.31	0.81 (0.74, 0.91)	0.00014
Mayo (Replication)						
All	808	1248	0.27	0.31	0.80 (0.70, 0.92)	0.0017
Men	546	603	0.26	0.31	0.80 (0.66, 0.95)	0.012
Women	262	645	0.27	0.31	0.83 (0.66, 1.04)	0.093

As the GWAS study performed by Treuitlein et al. that reported potential association of alcohol dependence with this SNP included only male cases, we also performed association analyses in sex-specific strata. These analyses suggest that the minor allele at this SNP is protective against alcohol dependence in both sexes (Table 2), having very similar effect size (i.e., odds ratio) in men and women.

Table 3 shows our results for association of alcohol dependence with the *ADH1C* SNP rs1614972, along with the results for this SNP in both the GWAS of Treutlein et al. [1], which originally reported the association that we intended to replicate, and the SAGE GWAS published by Bierut et al. [5]. The results of a meta-analysis summarizing the findings of these three studies are also shown, demonstrating strong evidence for association of rs1614972 with alcohol dependence (OR = 0.86, 95% CI = (0.80, 0.92), p = 4.7*10^−6^).

**Table 3:** Meta-analysis of the association of alcohol dependence with *ADH1C* SNP rs1614972.

Study	N	OR	95% CI	P-value
Treutlein et al. [1]	3792	0.81	(0.74,0.91)	0.00014
Bierut et al. (SAGE) [5]	3829	0.94	(0.84,1.04)	0.205
Mayo (current study)	2056	0.80	(0.70,0.92)	0.0017
**Meta-analysis**	9677	0.86	(0.80,0.92)	4.7*10^−6^

Association of alcohol dependence with other investigated SNPs was not replicated (see Table S1 in File S1 for complete set of results). Our analyses suggested possible association with SNP rs708006, which did not remain significant after Bonferroni correction (uncorrected p = 0.0074, p_corrected_ = 0.32). However, the estimated odds ratio in our study indicates a protective effect of the minor allele, while the association observed by Treutlein et al. indicated the minor allele was associated with increased risk of alcohol dependence. Thus, our results do not support the association observed by Treutlein et al. for this SNP.

Having obtained evidence for the association of rs1614972 with alcohol dependence consistent with the findings of Treutlein et al. [1], we further investigated whether this SNP is associated with alcohol consumption. Analysis of consumption in controls utilized an ordinal measure of consumption classified into rare, intermediate, and frequent drinking (see Methods section for details). Using a Spearman partial correlation analysis, we found that there was a small but marginally significant negative correlation (ρ = −0.07, p = 0.081) between the minor allele count at rs1614972 and consumption, indicating a potential association of the minor allele with lower (less frequent) alcohol consumption.

For the cases, we observed similar associations of the SNP genotypes with alcohol consumption as in the controls. Linear regression analysis of the cases from the alcohol withdrawal studies provided evidence of association between the rs1614972 genotype and average alcohol consumption, measured as average drinks per drinking day (p = 0.046). As in the analysis of controls, the minor allele of rs1614972 was associated with lower average alcohol consumption (regression coefficient beta = −1.44). Carriers of the minor allele also had a lower maximum number of drinks consumed in a 24-hour period, but this trend was not statistically significant (p = 0.26).

## Discussion

Treutlein and Rietschel [16] recently reviewed GWAS of alcohol addiction and noted that in these studies only two SNPs have received modest support for replication in a subsequent study. We investigated the association of alcohol dependence with 43 SNPs selected on the basis of results from the first two published GWAS of alcoholism. With the available sample size, which is comparable to sample sizes of the published GWAS of alcohol dependence, our study of 43 SNPs with prior evidence for association provides a powerful strategy for replication of earlier findings. Our replication study provides further evidence that the minor allele of rs1614972 in the alcohol dehydrogenase 1C gene (*ADH1C*) is associated with decreased risk of alcohol dependence. We extended this finding by demonstrating that there is a trend toward lower alcohol consumption associated with the minor allele at this SNP. This trend was observed both in the sample of alcohol-dependent subjects as well as independently in the non-alcoholic controls.

Alcohol dehydrogenase (ADH) and aldehyde dehydrogenase (ALDH) are the primary enzymes involved in alcohol metabolism. ADH genes, which encode different forms of ADH, are located in a gene cluster on chromosomal region 4q [17]. Certain alleles encoding ADH enzymes with higher activity have been shown to result in more rapid conversion of alcohol to acetaldehyde and have a protective effect on the risk of alcoholism. A number of studies have reached consistent replicated results suggesting the association of variants in ADH genes with alcohol use related phenotypes [2], [4], [17], [18], [19]. By extending the sample from the GWAS of Treutlein et al., a recent genome-wide association study obtained genome-wide significant evidence for association between alcohol dependence and a variant in the ADH gene cluster region [20].

SNP rs1614972 is an intronic SNP in low-moderate LD with rs1693482 and rs698 (r^2^ = 0.29–0.31), two non-synonymous SNPs also known as Arg272Gln and Ile350Val, respectively. These SNPs, which define the frequently studied *ADH1C* *1/*2 haplotypes, have been reported to be associated with risk of alcohol dependence and with alcohol consumption [21], [22], [23]. Martinez et al. found that rs1693482 and rs698, as well as a rare non-synonymous *ADH1C* SNP, rs283413, are associated with decreased alcohol metabolic rates resulting in significant delays in reaction and an increase in time of motor reaction even at alcohol blood concentrations under 500 mg per liter [19]. A study of an Irish sample [21] found association of alcohol dependence with rs1693482 and marginal evidence of association with rs698, but no evidence of association with rs1614972. Further studies are needed to determine whether rs1614972 plays a functional role in alcohol dependence risk, or is associated with the trait because it is in LD with other functional SNPs in *ADH1C*. Moreover, because of strong LD between SNPs in *ADH1C* and the *ADH1B* gene located near *ADH1C* [17], it is possible that the association is due to the effect of variants in *ADH1B*.

The SAGE study did not find evidence of association of SNP rs1614972 with alcohol dependence [5]. This may be due to differences in characteristics of cases between the different genetic studies of alcohol dependence. The SAGE sample consisted of subjects from three different studies including a study of alcoholism, a study of nicotine dependence, and a study of cocaine dependence. Thus, although all cases were alcohol dependent by DSM-IV criteria, many were initially recruited because of other substance dependencies. This sample may, therefore, have higher rates of multi-substance dependence, perhaps leading to lower power to detect genes that predispose specifically to alcohol-dependence, such as the ADH genes.

We did not replicate other SNPs with marginal evidence of association in the prior GWAS of alcohol dependence that we investigated. The SAGE study [5] found modest evidence of replication for rs13160562 (p = 0.03) in the *ERAP1* gene that was first reported to be associated with alcohol dependence by Treutlein et al. [1]. Our study did not provide further evidence supporting the association of this SNP with alcohol dependence (p = 0.78). Numerous factors may have contributed to the lack of replication of most results between these studies, including the fact that the earlier GWAS results did not reach statistical significance, and perhaps do not represent true associations. However, differences between studies, especially in case definition and recruitment strategies, may have also reduced chances of replication as a result of considerable phenotypic heterogeneity. For example, the GWAS (discovery) stage of the Treutlein et al. [1] study included only early age-of-onset male cases. This group may represent a specific subtype of alcohol addiction with unique genetic determinants. We were unable to run a subgroup analysis of our data resembling this study, as we did not have an adequate number of cases with documented early age-of-onset of alcohol dependence.

Our study was intended to provide independent replication for SNPs with marginal evidence of association with alcohol dependence in prior GWAS. Meta-analysis of genetic association study results can strengthen association findings, and is thus sometimes used in the context of replication studies [24]. However, when combining results for top hits from a discovery GWAS with results of a study that attempts replication, care must be taken in interpreting the findings, because top hits selected from underpowered GWAS, particularly top hits that did not reach genome-wide significance levels in the discovery study, likely have inflated odds ratio estimates in the discovery study due to winner’s curse [24]. Nevertheless, meta-analysis can be a powerful technique for combining discovery GWAS results with subsequent candidate-gene replication, as long as a stringent genome-wide significance threshold is applied to the meta-analysis results, as was done, for example, by Esserlind et al. [25], who reported meta-analysis odds ratio estimates only for SNPs that met genome-wide significance criteria in the combined analysis (including three SNPs with significant evidence for replication, after multiple testing correction, and one SNP without significant evidence for replication in their study, but with genome-wide significant results in the discovery data and other subsequent replication studies). However, when the results do not replicate in a similar-sized sample, a meta-analysis combining the discovery and replication results is likely to produce inflated odds ratio estimates. As most of the SNPs that we investigated were not genome-wide significant in the original discovery studies, and did not achieve even nominal significance in our replication study, often showing point estimates of effects in opposite direction from the initial discovery study, we do not present meta-analysis estimates for all SNPs investigated in this manuscript, and rather focus on the independent replication results. Nevertheless, for the two SNPs that showed genome-wide significant results in the combined GWAS and replication analysis within the Treutlein et al. [1] study (rs1344694 and rs7590720) we combined our results with those reported for the pooled sample of Treutlein via meta-analysis. For both of these SNPs, addition of our data to that of Treutlein led to a decrease in the significance of the findings, with the meta-analysis providing non-significant evidence of association at the genome-wide threshold of 5*10^−8^ (results not shown). As more genetic studies of alcohol dependence are performed (both replication studies and additional GWAS), meta-analysis of all available results will be an important step that may lead to identification of new genome-wide significant findings.

Limitations of this study include low coverage of variation in genes of interest. As the goal of this study was to replicate specific SNP associations suggested by prior GWAS of alcohol dependence, we genotyped only one or two SNPs per gene. However, for the replicated signal (rs1614972), it would be interesting to investigate associations with other SNPs in the gene. Thus, additional studies should investigate the association of other *ADH1C* SNPs with alcohol dependence and related phenotypes, as well as the potential functional role of the rs1614972 SNP, and SNPs in LD with it including the non-synonymous SNPs rs1693482 and rs698. Furthermore, investigation of other SNPs in the top genes identified by prior GWAS may identify important associations with alcohol dependence. For example, although here we did not replicate the rs13273672 SNP association in the *GATA4* gene first reported in the GWAS of Treutlein et al [1], in a recent study we were able to demonstrate association of alcohol dependence with *GATA4* using a gene-level test [26].

Another limitation is the lack of data on secondary alcohol-related phenotypes collected in a consistent manner for all subjects, which limited the possible secondary analyses of additional phenotypes for alcohol-dependence associated SNPs. Difficulties with standardizing phenotypes for data collected as part of multiple studies is a well-known problem. Our study included alcohol-dependent cases from Mayo Clinic’s DNA Repository for Genomic Studies of Addiction, as well as from ongoing studies of genetic predictors of severe alcohol withdrawal [6], [7], and a liver transplant study. Because the cases had been recruited as part of different protocols, consistent tools were not utilized for data collection, leading to phenotypic heterogeneity in the analyzed data. Due to the limited availability of consistently collected data, we were unable to evaluate genetic association with other consumption measures. Further studies of the role of *ADH1C* in alcohol use disorders should consider other alcohol-use related phenotypes.

The lack of genome-wide SNP data for cases in our study precluded the application of principal component analysis to control for population stratification. Many candidate gene studies rely on self-reported ancestry to control for population stratification, an approach that has been shown to be reliable in genetic studies [27], [28]. As our study included only self-reported Caucasian cases and controls, recruited at a single site, population stratification is not expected to strongly confound results. Nevertheless, we genotyped a set of ancestry informative SNPs [13] that allowed us to verify self-reported race using STRUCTURE analysis [14]. Analyses adjusted for possible population stratification by inclusion of covariates representing ancestry proportions provided results almost identical to the unadjusted analyses.

This study utilized a previously genotyped set of controls. Use of such samples of convenience is increasingly recognized as an efficient approach to genetic studies (see for example [29], [30]). The genotyping of one common CEPH control with both the cases and the controls provided additional reassurance of genotype concordance across the two platforms used in our study.

Finally, although our candidate gene study was based on a fairly large sample, comparable to the sample sizes used in the published GWAS of addiction, it still offered limited power to detect small effect sizes. In particular, the study was not well powered to detect odds ratios below 1.3. Thus, it remains possible that some of the variants that we investigated are in fact associated with alcohol dependence, but with relatively small effects.

In conclusion, data presented here provide additional support for the association of SNP rs1614972 in the *ADH1C* gene with alcohol dependence, as well as with alcohol consumption among alcoholics and non-alcoholics. Further studies should investigate the association of other polymorphisms in this gene with alcohol use related phenotypes, in particular SNPs that are in LD with rs1614972. Functional studies should be performed to determine which of the SNPs in this region may be causally associated with alcohol dependence, via a direct functional effect.

## Supporting Information

Supplemental File S1 **Table S1**: Association Test Results for All SNPs. **Table S2:** Ancestry Informative SNPs. **Figure S1**: Plot of STUCTURE analysis results including the study subjects (cases and controls) and HapMap samples. 1 = HapMap samples (red = YRI, blue = CHB, green = CEU), 2 = Controls (teal = self-reported non-Caucasian, black = self-reported Caucasian), 3 = Cases (teal = self-reported non-Caucasian, black = self-reported Caucasian). The two circled subjects represent the self-reported Caucasian subjects (one case and one control) that were excluded because the structure analysis indicated >30% African ancestry. Self-reported minorities (shown in teal) were also excluded.
(DOC)
