# Supplementary material for: Replication of Genome Wide Association Studies of Alcohol Dependence: Support for Association with Variation in ADH1C
Source: PLoS One. 2013 Mar 13;8(3):e58798. doi: 10.1371/journal.pone.0058798 (PMC3596339; doi:10.1371/journal.pone.0058798)
Supplement: Supplemental File S1 — Table S1: Association Test Results for All SNPs. Table S2: Ancestry Informative SNPs. Figure S1: Plot of STUCTURE analysis results including the study subjects (cases and controls) and HapMap samples. 1 = HapMap samples (red = YRI, blue = CHB, green = CEU), 2 = Controls (teal = self-reported non-Caucasian, black = self-reported Caucasian), 3 = Cases (teal = self-reported non-Caucasian, black = self-reported Caucasian). The two circled subjects represent the self-reported Caucasian subjects (one case and one control) that were excluded because the structure analysis indicated >30% African ancestry. Self-reported minorities (shown in teal) were also excluded. (DOC) [file pone.0058798.s001.doc]

**Supplemental File Materials**

**Supplemental File, Table S1: Association Test Results for All SNPs**

| SNP | CHR | BP | gene | Major Allele | Minor Allele | MAF cases | MAF controls | OR (95% CI) | P | study with prior association |
| --- | --- | --- | --- | --- | --- | --- | --- | --- | --- | --- |
| rs4478858^b^ | 1 | 31656512 | *SERINC2* | T | C | 0.415 | 0.409 | 1.03 (0.91,1.17) | 0.63 | SAGE GWAS |
| rs497159^c^ | 1 | 55326142 | *USP24* | C | T | 0.157 | 0.157 | 1.01 (0.85,1.20) | 0.94 | SAGE GWAS |
| rs1165238 | 1 | 55418594 | *USP24* | T | C | 0.197 | 0.198 | 1.00 (0.85,1.16) | 0.95 | SAGE GWAS |
| rs11579497 | 1 | 194328334 | *KCNT2* | G | A | 0.192 | 0.186 | 1.04 (0.89,1.22) | 0.60 | German GWAS |
| rs12036795 | 1 | 194356441 | *KCNT2* | A | C | 0.308 | 0.302 | 1.03 (0.90,1.18) | 0.65 | German GWAS |
| rs1344694 | 2 | 216601882 | *PECR* | G | T | 0.337 | 0.329 | 1.05 (0.91,1.20) | 0.51 | German GWAS |
| rs7590720 | 2 | 216606903 | *PECR* | A | G | 0.295 | 0.284 | 1.06 (0.92,1.22) | 0.41 | German GWAS |
| rs705648 | 2 | 216642037 | *PECR* | T | C | 0.251 | 0.247 | 1.05 (0.91,1.22) | 0.51 | German GWAS |
| rs1201284 | 3 | 178290008 | *TBL1XR1* | G | A | 0.155 | 0.148 | 1.06 (0.89,1.26) | 0.52 | German GWAS |
| rs1209035 | 3 | 178310621 | *TBL1XR1* | T | C | 0.102 | 0.099 | 1.03 (0.84,1.27) | 0.78 | German GWAS |
| rs1614972 | 4 | 100477178 | *ADH1C* | C | T | 0.265 | 0.311 | 0.80 (0.70,0.92) | **0.0017** | German GWAS |
| rs10068363^a,b^ | 5 | 62606652 | *ISCA1L* | A | G | failed | 0.136 | NA | NA | SAGE GWAS |
| rs2169520^a,b^ | 5 | 62634209 | *ISCA1L* | T | C | 0.204 | 0.199 | 1.03 (0.88,1.21) | 0.69 | SAGE GWAS |
| rs10514959^a,b^ | 5 | 62639403 | *ISCA1L* | A | C | 0.124 | 0.115 | 1.10 (0.91,1.33) | 0.35 | SAGE GWAS |
| rs1462460^a,b^ | 5 | 62649397 | *ISCA1L* | G | A | 0.116 | 0.109 | 1.08 (0.88,1.31) | 0.47 | SAGE GWAS |
| rs7714594^a,b^ | 5 | 62690438 | *ISCA1L* | C | A | 0.149 | 0.143 | 1.05 (0.88,1.25) | 0.59 | SAGE GWAS |
| rs6882052^a,b^ | 5 | 62702058 | *ISCA1L/HTR1A* | G | T | 0.261 | 0.274 | 0.94 (0.82,1.09) | 0.41 | SAGE GWAS |
| rs13362120 | 5 | 96096950 | *CAST* | T | C | 0.305 | 0.303 | 1.04 (0.90,1.19) | 0.60 | German GWAS |
| rs13160562 | 5 | 96137127 | *CAST/ERAP1* | G | A | 0.290 | 0.285 | 1.03 (0.89,1.19) | 0.69 | German GWAS |
| rs1864982 | 5 | 146301016 | *PPP2R2B* | G | T | 0.123 | 0.139 | 0.86 (0.72,1.04) | 0.12 | German GWAS |
| rs708006 | 6 | 37042222 | *MTCH1/PI16* | C | A | 0.142 | 0.173 | 0.79 (0.66,0.94) | 0.0074 | German GWAS |
| rs4714028 | 6 | 37063866 | *MTCH1* | C | A | 0.430 | 0.412 | 1.08 (0.95,1.22) | 0.24 | German GWAS |
| rs6902771 | 6 | 152199574 | *ESR1* | C | T | 0.470 | 0.476 | 0.98 (0.87,1.11) | 0.73 | German GWAS |
| rs17142876^b^ | 7 | 120063197 | *KCND2* | A | G | 0.103 | 0.086 | 1.21 (0.98,1.50) | 0.0745 | SAGE GWAS |
| rs728115^b^ | 7 | 120092385 | *KCND2* | G | A | 0.108 | 0.091 | 1.21 (0.98,1.48) | 0.0729 | SAGE GWAS |
| rs729302 | 7 | 128356196 | *IRF5* | A | C | 0.308 | 0.317 | 0.96 (0.84,1.10) | 0.54 | German GWAS |
| rs13273672 | 8 | 11649790 | *GATA4* | T | C | 0.339 | 0.325 | 1.07 (0.94,1.22) | 0.33 | German GWAS |
| rs1487814 | 11 | 21907061 | *TMEM16E* | T | C | failed | 0.468 | NA | NA | German GWAS |
| rs1426153^a,b^ | 11 | 124675849 | *PKNOX2* | A | G | 0.157 | 0.166 | 0.94 (0.79,1.11) | 0.46 | SAGE GWAS |
| rs750338^a,b^ | 11 | 124677803 | *PKNOX2* | A | G | 0.204 | 0.206 | 0.98 (0.84,1.15) | 0.84 | SAGE GWAS |
| rs12284594^a,b^ | 11 | 124685063 | *PKNOX2* | A | G | 0.155 | 0.164 | 0.93 (0.79,1.11) | 0.43 | SAGE GWAS |
| rs4768085 | 12 | 43941331 | *TMEM16F* | C | A | 0.057 | 0.051 | 1.12 (0.85,1.47) | 0.44 | German GWAS |
| rs7138247 | 12 | 43956020 | *TMEM16F* | A | G | 0.064 | 0.057 | 1.12 (0.86,1.45) | 0.40 | German GWAS |
| rs7965430 | 12 | 43996497 | *TMEM16F* | G | A | 0.061 | 0.057 | 1.07 (0.82,1.40) | 0.60 | German GWAS |
| rs1800973 | 12 | 68030281 | *LYZ/YEATS4* | C | A | 0.068 | 0.069 | 0.99 (0.77,1.27) | 0.93 | German GWAS |
| rs4761605 | 12 | 93253708 | *CCDC41* | C | T | 0.120 | 0.118 | 1.02 (0.84,1.24) | 0.83 | German GWAS |
| rs7138291 | 12 | 93265837 | *CCDC41* | T | C | 0.117 | 0.117 | 1.01 (0.83,1.22) | 0.95 | German GWAS |
| rs36563 | 14 | 70422401 | *PCNX* | G | T | 0.162 | 0.163 | 0.99 (0.84,1.18) | 0.94 | German GWAS |
| rs1012259 | 16 | 5892910 | *A2BP1* | G | A | 0.381 | 0.375 | 1.03 (0.90,1.17) | 0.70 | German GWAS |
| rs11077030 | 16 | 6300618 | *A2BP1* | A | G | 0.062 | 0.053 | 1.18 (0.90,1.55) | 0.22 | German GWAS |
| rs17572451 | 16 | 76764057 | *WWOX* | A | G | 0.093 | 0.103 | 0.90 (0.73,1.11) | 0.32 | SAGE GWAS |
| rs11640875 | 16 | 81278925 | *CDH13* | G | A | 0.330 | 0.343 | 0.95 (0.83,1.09) | 0.45 | German GWAS |
| rs12388359 | 23 | 10191691 | *CLCN4* | G | T | 0.127 | 0.133 | 0.97 (0.83,1.12) | 0.65 | German GWAS |
| rs11095531 | 23 | 10243239 | *CLCN4* | A | G | 0.210 | 0.208 | 1.01 (0.89,1.14) | 0.91 | German GWAS |
| rs2214261 | 23 | 10265329 | *CLCN4* | G | A | 0.158 | 0.151 | 1.06 (0.92,1.22) | 0.42 | German GWAS |
| rs12392447 | 23 | 153377545 | *UBL4A/SLC10A3* | G | A | failed | 0.119 | NA | NA | German GWAS |

^a^SNPs in regions or genes that were reported to be associated with alcohol dependence in the SAGE data by Bierut et al. [5]

^b^SNPs in regions or genes that were reported to be associated with alcohol dependence in the SAGE data by Zuo et al. [12]

^c^SNP rs497159 was selected as a proxy for rs615652 that had p<10^‑4^ in our analysis of the SAGE data, but had not been genotyped in the available controls for this study

**Supplemental File, Table S2: Ancestry Informative SNPs**

| SNP | Chr | BP position | Major allele | Minor allele | MAF cases | MAF controls |
| --- | --- | --- | --- | --- | --- | --- |
| rs10007810 | 4 | 41249121 | G | A | 0.220 | 0.218 |
| rs10108270 | 8 | 4178201 | C | A | 0.289 | 0.282 |
| rs1040045 | 6 | 4692158 | A | G | 0.250 | 0.258 |
| rs11652805 | 17 | 60417613 | T | C | 0.160 | 0.168 |
| rs2416791 | 12 | 11592755 | G | A | 0.102 | 0.123 |
| rs260690 | 2 | 108946170 | A | C | 0.067 | 0.063 |
| rs3737576 | 1 | 101482151 | T | C | failed | 0.054 |
| rs3784230 | 14 | 104750100 | A | G | failed | 0.407 |
| rs4891825 | 18 | 66018643 | A | G | 0.090 | 0.090 |
| rs4908343 | 1 | 27804285 | A | G | 0.157 | 0.177 |
| rs6422347 | 5 | 177795689 | T | C | 0.081 | 0.064 |
| rs6451722 | 5 | 43747135 | G | A | 0.199 | 0.218 |
| rs6548616 | 3 | 79482265 | T | C | 0.286 | 0.278 |
| rs7554936 | 1 | 149389113 | T | C | 0.347 | 0.344 |
| rs7657799 | 4 | 105594872 | T | G | 0.024 | 0.018 |
| rs772262 | 12 | 54450001 | G | A | 0.077 | 0.074 |
| rs7997709 | 13 | 33745737 | T | C | 0.052 | 0.049 |
| rs870347 | 5 | 6898035 | A | C | 0.073 | 0.065 |
| rs9319336 | 13 | 26522356 | T | C | 0.056 | 0.053 |
| rs9522149 | 13 | 110625168 | C | T | 0.263 | 0.269 |
| rs9530435 | 13 | 74891888 | C | T | 0.155 | 0.174 |
| rs9845457 | 3 | 137397166 | A | G | 0.374 | 0.384 |
| rs10236187 | 7 | 139093846 | A | C | failed | 0.035 |
| rs12629908 | 3 | 122005406 | G | A | 0.052 | 0.047 |
| rs1325502 | 1 | 42132857 | G | A | 0.165 | 0.159 |
| rs13400937 | 2 | 79718431 | T | G | 0.273 | 0.286 |
| rs2125345 | 17 | 71293786 | T | C | 0.308 | 0.305 |
| rs3907047 | 20 | 53434321 | T | C | 0.051 | 0.054 |
| rs3943253 | 8 | 13403871 | A | G | 0.075 | 0.076 |
| rs4746136 | 10 | 74971000 | G | A | 0.138 | 0.150 |

**Supplemental Figure S1: Plot of STUCTURE analysis results including the study subjects (cases and controls) and HapMap samples**. 1=HapMap samples (red=YRI, blue=CHB, green=CEU), 2=Controls (teal=self-reported non-Caucasian, black=self‑reported Caucasian), 3=Cases (teal=self-reported non-Caucasian, black=self-reported Caucasian). The two circled subjects represent the self-reported Caucasian subjects (one case and one control) that were excluded because the structure analysis indicated >30% African ancestry. Self-reported minorities (shown in teal) were also excluded.
